# Immunologic Insights on the Membrane Proximal External Region: A Major Human Immunodeficiency Virus Type-1 Vaccine Target

**DOI:** 10.3389/fimmu.2017.01154

**Published:** 2017-09-19

**Authors:** Luis M. Molinos-Albert, Bonaventura Clotet, Julià Blanco, Jorge Carrillo

**Affiliations:** ^1^IrsiCaixa AIDS Research Institute, Institut de Recerca Germans Trias i Pujol (IGTP), Germans Trias i Pujol University Hospital, Barcelona, Spain; ^2^Universitat de Vic – Universitat Central de Catalunya, Barcelona, Spain

**Keywords:** human immunodeficiency virus type-1, broadly neutralizing antibodies, membrane proximal external region, B-cells, polyreactivity, membrane interaction, immunization, immunogens

## Abstract

Broadly neutralizing antibodies (bNAbs) targeting conserved regions within the human immunodeficiency virus type-1 (HIV-1) envelope glycoprotein (Env) can be generated by the human immune system and their elicitation by vaccination will be a key point to protect against the wide range of viral diversity. The membrane proximal external region (MPER) is a highly conserved region within the Env gp41 subunit, plays a major role in membrane fusion and is targeted by naturally induced bNAbs. Therefore, the MPER is considered as an attractive vaccine target. However, despite many attempts to design MPER-based immunogens, further study is still needed to understand its structural complexity, its amphiphilic feature, and its limited accessibility by steric hindrance. These particular features compromise the development of MPER-specific neutralizing responses during natural infection and limit the number of bNAbs isolated against this region, as compared with other HIV-1 vulnerability sites, and represent additional hurdles for immunogen development. Nevertheless, the analysis of MPER humoral responses elicited during natural infection as well as the MPER bNAbs isolated to date highlight that the human immune system is capable of generating MPER protective antibodies. Here, we discuss the recent advances describing the immunologic and biochemical features that make the MPER a unique HIV-1 vulnerability site, the different strategies to generate MPER-neutralizing antibodies in immunization protocols and point the importance of extending our knowledge toward new MPER epitopes by the isolation of novel monoclonal antibodies. This will be crucial for the redesign of immunogens able to skip non-neutralizing MPER determinants.

## Introduction

### An Apparently Easy Vaccine Target

The human immunodeficiency virus type-1 (HIV-1) envelope glycoprotein (Env) is the sole viral antigen exposed on the virion surface. Env is synthetized as a precursor gp160 glycoprotein that will yield after cleavage a mature complex constituted by the non-covalent association of three gp120 (surface) and three gp41 (transmembrane) subunits, resulting in a heavily glycosylated trimer of heterodimers (1–5). Env determines the process of HIV-1 entry into the target cell that will lead to the fusion of the viral and host cell membranes (6). This process initiates with the high affinity interaction between gp120 and the CD4 molecule on the surface of target cells. This interaction promotes a series of conformational changes that transiently expose the gp120 coreceptor binding site allowing the gp120 attachment to the CCR5 or CXCR4 chemokine receptors (7–9). Coreceptor ligation triggers structural rearrangements in gp41 that permit the initiation of viral fusion. The gp41 fusion peptide (FP) inserts into the target cell membrane accounting for a short-life prehairpin fusion intermediate in which both cellular and viral membranes are connected by an extended conformation of gp41. Next, alpha-helical domains HR1 and HR2 of each gp41 monomer are folded back together to generate a 6-helix bundle conformation that brings both target cell and viral membranes closer to finally produce the membrane merge (10, 11). During this process both FP and the membrane proximal external region (MPER) play a crucial role in membrane destabilization (12).

Given its exposure on the virion surface and its role in viral infectivity, Env is the main target of HIV-1 protective humoral responses. The elicitation of Env broadly neutralizing antibodies (bNAbs), defined as those capable of neutralize the wide viral diversity, is one of the main goals for a successful HIV-1 vaccine (13). The notion that the human immune system is capable of producing HIV-1 bNAbs has been established by two pieces of evidence: (i) the identification of such immune responses in sera from HIV-1 infected individuals and (ii) the isolation of monoclonal bNAbs from these individuals (14, 15). These naturally induced bNAbs allowed the identification of conserved Env regions that helped researchers to delineate an HIV-1 Env vulnerability map. The study of bNAbs and the epitopes targeted by them are contributing enormously to our understanding of the HIV-1 humoral response as well as to the rational design of immunogen candidates (14, 16).

Whereas a big collection of bNAbs against gp120 Env subunit has been generated, a limited number has been also isolated against the less exposed gp41 subunit (17). Although neutralizing antibodies targeting the HR1 alpha-helical region have been described (18–20), the MPER is the major gp41 neutralizing determinant (21, 22). This highly conserved and unusual tryptophan-rich motif is located adjacent to the viral membrane, covering the last C-terminal residues of the gp41 ectodomain (aa 660–683, HXB2 numbering) and connects the extracellular portion of Env with the TM domain (23, 24). The importance of the MPER on Env functionality was highlighted by analysis of mutant viruses containing deletions, insertions or substitutions within this region (24–26). Substitution of the five MPER conserved tryptophan residues dramatically compromised the incorporation of gp41 into virions and, thus, blocked viral entry (24). Moreover, simple deletion of the W_666_-I_682_ spanning sequence completely abolished syncytium formation (27). These observations indicated that the MPER plays a major role in the HIV-1 Env-mediated fusion and viral infectivity, which is consistent with the high level of sequence conservation (23). The functional implications in viral infectivity, the high level of conservation and the lack of N-linked glycosylated residues, together with the discovery of potent and/or bNAbs targeting linear MPER sequences (2F5, 4E10, 10E8), all able to protect against viral challenge in non-human primates (NHP) (28–30), points that the elicitation of MPER-specific neutralizing responses by immunogen candidates is highly desirable (21, 22, 31). In addition, the MPER has a role in HIV-1 CD4-independent viral transcytosis at the epithelial barrier (32), where the conserved _662_ELDKWA_667_ gp41 sequence interacts with galactosyl ceramide receptors (33). Secretory IgA from cervicovaginal secretions of HIV-1 infected individuals are capable of blocking viral transcytosis via _662_ELDKWA_667_ sequence binding (34).

The MPER presents some immunological, physical, and structural, properties that impact directly on its immunogenicity, explaining the lower MPER neutralizing response of HIV-1 infected individuals comparing with other Env vulnerability regions (35, 36). Those include steric hindrance by gp120 and high hydrophobicity that makes the MPER to be partially embedded within the viral membrane (37). Structurally, the information regarding the native conformation of the MPER within the Env trimer is still limited (5), adding the challenge of developing an immunogen against a structurally ambiguous epitope. Finally, MPER-specific bNAbs show reactivity against self-antigens and host tolerance mechanisms have been suggested to influence the elicitation of MPER neutralizing responses (38).

Here, we discuss the properties that make the MPER both a unique as well as a challenging HIV-1 vaccine target; we review the MPER immune response during natural infection, the particular features of MPER bNAbs isolated and the different attempts to generate MPER-specific neutralizing antibodies by immunization within the last years. Although the results reflect a generalized failure, new insights into our knowledge have been achieved. The fact that other Env vulnerability sites have followed a similar path supports the notion that the MPER is still an HIV-1 vaccine target worth exploring (31).

## Isolation of MPER Neutralizing Antibodies

The strongest evidence supporting that the human immune system can develop a potent neutralizing MPER-specific response results from the isolation of monoclonal antibodies from HIV-infected individuals. From the naturally induced 2F5, 4E10, 10E8, z13, m66.6, and CH12 antibodies identified, three of them (2F5, 4E10, and 10E8) display a broadly neutralizing activity (28, 39–46). 2F5 and 4E10 are among the first HIV-1 bNAbs discovered. They were generated by electrofusion of peripheral blood mononuclear cells mixtures from different HIV-1 infected individuals (47). 2F5 targets the linear sequence _662_ELDKWA_667_ (39) within the N-terminal moiety of the MPER, where the central core _664_DKW_666_ is essential for neutralization, as demonstrated by alanine-scanning mutagenesis assays (48). 2F5 has a relatively high potency and was found to neutralize 57–67% of the viral isolates tested with an IC50 below 50 µg/mL (42, 49). However, HIV-1 subtype C viruses are usually 2F5-resistant due to a mutation in the central core epitope (DSW instead of DKW) (49–51). 4E10 targets the distal conserved tryptophan rich moiety located C-terminal to the 2F5 epitope which includes the sequence _671_NWFDIT_676_ and is extended toward C-terminal residues, where W672, F673, I675, T676, L679, and W680 have the most significant contacts with the antibody (43). Although presenting a moderate potency, 4E10 displays a remarkable breadth against 98–100% of the viral isolates, depending of the panel tested, with an IC50 below 50 µg/mL (49, 52). Further characterization of 2F5 and 4E10 antibodies has shown reduced potency of both antibodies, against transmitted-founder viruses (T/F IMC) or against replicating viruses obtained from primary lymphocytes when compared with pseudovirus obtained in 293 T cells (53–56). Despite these potential limitations, both 2F5 and 4E10 were shown to protect against viral challenge in NHP (28, 29) and their administration into human recipients showed no major clinical complications (57).

In order to delineate a complete map of HIV-1 neutralizing determinants, starting in 2009, a substantial effort has been made on the isolation of new bNAbs. The development of high-throughput analysis of single memory B cells and the use of fluorescently labeled Env-based protein probes to isolate antigen specific B cells (58–60) contributed enormously to the discovery of new HIV-1 neutralizing antibodies. In this context, the discovery in 2012 of the monoclonal antibody 10E8 recovered the interest toward the MPER region as a major vaccine target (42). 10E8 neutralized 98% of a panel of 181 pseudovirus with an IC50 below 50 µg/mL, showing a mean IC50 for sensitive viruses of 0.25 µg/mL, whereas mean IC50 values for 4E10 and 2F5 were 1.3 and 1.92 µg/mL, respectively (42). Interestingly, 72% of the panel was neutralized by 10E8 with an IC50 below 1 µg/mL, comparing with 37 and 16% for 4E10 and 2F5, respectively (42). Therefore, 10E8 could neutralize with a far greater potency and breadth than previously discovered anti-MPER bNAbs 2F5 and 4E10, and was comparable with some of the most potent HIV-1 bNAbs like VRC01 or PG9/PG16 (15). Notably, 10E8 was also reported to protect against viral challenge *in vivo* (30).

Interestingly, 2F5, 4E10, and 10E8 antibodies are IgG3 (42, 61); however, the role of this IgG subclass in the neutralizing properties of these antibodies, if any, remains elusive. Although IgG1 and IgG3 are the predominant antibodies elicited against viral antigens (62), both subclasses show important differences. IgG3 shows higher affinity for Fcγ receptors than IgG1, a shorter half-life and a long highly flexible hinge region which has been suggested to be crucial to facilitate the access of these antibodies to the MPER and mediate their neutralizing activity (63, 64). However, it is still unclear whether an IgG3 background is absolutely required, since anti-MPER neutralizing responses have been identified in the non-IgG3 fraction of some HIV-infected individuals (65), and a change to IgG1 did not affect the neutralizing activity of 2F5 and 4E10 antibodies (61, 66). In this context, anti-MPER bNAbs could have been specifically generated from germline precursors preferentially undergoing IgG3 class switching (67) and, in some cases, after affinity maturation and antigen selection by somatic hypermutation, switching to a more downstream IgG subclasses, such as IgG1, by sequential class switching recombination (68). Because IgG3 is one of the less represented IgG subclasses, with the shortest half-life in plasma and IgG3-dominant humoral responses are uncommon (63), elucidating whether this IgG subclass is required for the development of anti-MPER bNAbs, might be crucial to define immunization strategies aimed to generate effective long-lasting anti-MPER responses.

Independently of their origin, all these antibodies are the result of a long process of affinity maturation and are highly mutated with an unusually long and hydrophobic IgH complementary determining region 3 (CDR H3) (42, 69, 70). Notably, these antibodies share a common neutralization mechanism in which the interaction of the hydrophobic CDR H3 apex with the membrane seems to be essential (see next section) (71, 72). Accordingly, autoreactivity/polyreactivity are odd characteristics of 2F5 and 4E10 antibodies. Initially, 10E8 was reported to be non-polyreactive but subsequent studies suggested that 10E8 needs to bind membrane lipids, especially cholesterol, to mediate neutralization (42, 73, 74).

Depending on the bound antibody, the MPER can acquire a particular conformation. Crystal structures of 2F5 in complex with an MPER peptide showed that the core motif DKW forms a type 1 β-turn structure (75). Contrary, the MPER in complex with 4E10 was found to form an α-helical conformation from D674 to K683 (70, 76). Recently, the crystal structure of 10E8 bound to an scaffolded MPER construct revealed that the full epitope of 10E8 is composed of both MPER and lipids (74). Encouragingly, the frequency of 10E8-like antibodies in HIV-infected individuals seemed to be superior to 2F5 or 4E10 specificities in the cohort where 10E8 was isolated (42).

Very recently, a new lineage of distal MPER-specific bNAbs, designated as DH511, was isolated from memory B-cells and plasma of an HIV-infected donor (67). DH511 lineage presented long CDR H3 loops of 23 to 24 aminoacids, an VH and VL somatic mutation rate of 15–22 and 14–18%, respectively, and was derived from the same heavy chain germline gene family as 10E8 (VH 3–15). Similarly to 2F5, 4E10, and 10E8, DH511 clonal lineage presented an IgG3 isotype. Interestingly, the most potent mAb of this clonal lineage, DH511.2, neutralized 206 out of 208 pseudovirus of a geographically and genetically diverse panel with a median IC50 of 1 µg/mL, being slightly more broad but less potent than 10E8 (67).

## Lipid Binding and the Concern of Polyreactivity

### MPER and Lipids

Biophysical models suggest that the MPER acquires an alpha-helical conformation partially embedded into the viral membrane, constituted by two independent domains separated by a flexible hinge (37, 77). These two segments showed to present different membrane-interacting properties. The C-terminal domain remains embedded into the membrane, whereas the N-terminal domain is more exposed (37, 77–79). The high tryptophan content is likely responsible of the MPER potential to interact with and destabilize lipid membranes (80, 81). According to its amphiphilic characteristics, hydrophobic residues remain buried into the membrane whereas the most polar ones are solvent-exposed (37). Of note, the MPER topology depends on the membrane context where it is presented (82, 83) and membrane lipids such as cholesterol and sphingomyelin can modulate the capacity of the MPER to destabilize membranes (82, 83). MPER and cholesterol interactions are further supported by the existence of the sequence 679-LWYIK-683 located at the C-terminus which was identified as a cholesterol recognition amino acid consensus motif (84). This motif seems to play an important role during the incorporation of Env into the virion, stabilizing the trimer complex (22).

### Neutralization Mechanisms and the Importance of Membrane Interaction

Antibody binding to a precise peptide sequence is necessary but not sufficient to achieve MPER-dependent antibody neutralization. Accordingly, z13e1 or 13H11 antibodies overlap the sequences bound by 4E10 and 2F5 respectively with similar affinities but displaying a far low neutralization potency (44, 85). MPER bNAbs show an enrichment of their long CDR H3 loops in hydrophobic residues that seem to be important for their neutralization capability (48, 86, 87). Whereas some residues of the CDRs are important for binding to the peptidic epitope, the most hydrophobic loops interact directly with membrane lipids (71, 72, 87). SPR-based studies demonstrated that whereas anti-MPER bNAbs bind to a peptide sequence following a Langmuir curve model, binding against peptide-membrane complexes follow a two steps (encounter-docking) model. First, the antibody attaches to the lipid membrane through its long hydrophobic CDR H3 and concentrates within the proximity of the MPER epitope to subsequently bind to the prehairpin intermediate of gp41, once the conformational change takes place (71, 72). This mechanism facilitates the accessibility of the antibody to its epitope, overcoming the poor exposure of the MPER and takes advantage of its close proximity to the viral membrane. Of note, upon binding, 2F5 or 4E10 promote an MPER conformational change, due to the extraction of the membrane-embedded epitope (37, 77).

Interestingly, the 2F5 antibody was predicted to bind lipids via CDRL1 and CDRH3 (88) and lipid binding sites were recently determined for 4E10 and 10E8 by X-ray crystallography (74, 89). 4E10 was shown to interact specifically with phosphatidic acid, phosphatidylglicerol and glycerol phosphate by using the CDR H1 and CDR H3 loops to bind polar head and hydrophobic tail groups respectively (89). In a second study, 10E8 lipid binding site was identified at the proximity of CDR L1 and CDR H3 loops (74). Therefore, the full epitope of MPER bNAbs is constituted by both peptide residues and membrane lipids. Notably, neutralizing activity of an anti-MPER single-chain bivalent llama antibody induced by immunization was also dependent of the hydrophobic CDR H3 apex without being involved in peptide recognition (87). Membrane interaction, thus, seems to play a major role in the neutralization mechanism of MPER bNAbs (26, 37, 72, 73, 77, 86).

The widely described importance of the membrane in MPER structure and functionality of the specific bNAbs suggest a role of lipids as a natural scaffold shaping the MPER structure. In this regard it is likely that lipids participate in the selection of germline precursors of bNAbs, pointing their relevance for immunogen design. Therefore, the generation of neutralizing anti-MPER responses may require its presentation within a membrane environment to properly present neutralizing determinants and to implement lipid cross-reactivity. The role of membrane lipids over MPER immunogenicity is, thus, a relevant issue currently being evaluated in immunization studies.

### Binding to Self-Antigens: A Major Roadblock for MPER Neutralizing Antibodies?

Reactivity with self-antigens was suggested to explain the failure of generating MPER neutralizing antibodies by immunization as well as their low frequencies during natural infection (38, 90, 91). Gp41 antibodies generated during acute infection are usually derived from polyreactive antibodies whose precursors cross-react with antigens from intestinal microbiota (92–94). In 2005, polyspecific binding of 4E10 and 2F5 mAbs to cardiolipin and other anionic phospholipids was reported (90). Furthermore, conserved host antigens bound by 2F5, 4E10 and 10E8 have been also identified (95, 96). 2F5 binds to the enzyme kinureninase (KYNU), which contains the identical sequence (ELDKWA) of the 2F5 epitope, and is highly conserved between different mammal species. 4E10 binds to splicing factor-3b subunit-3 and type I inositol triphosphate (IP_3_R1) (95) and, although initially described as non-autoreactive, 10E8 recognize the FAM84A protein (96). Collectively, these findings suggested that immunological tolerance might be involved in HIV-1 evasion of immune responses since autoreactive B-cells that cross-react with MPER sequences might be impaired in the naive repertoire (91, 97).

This hypothesis was tested by monitoring B-cell development in knock-in (KI) mice models carrying the same V(D)J rearrangements as mature bNAbs 2F5 and 4E10. These models showed a normal early B cell development but exhibited a blockade in the transition of pre-B to immature IgM+ B cells, which is defined by the first tolerance checkpoint (98–101). B-cell central tolerance takes place in the bone marrow (BM) and abrogates the development of autoreactive B-cells by several mechanisms such as clonal deletion or receptor edition (102). After that, some autoreactive B-cells can still egress from BM as anergic cells, which show a hyporesponder status and a reduced lifespan. However, in special circumstances anergic B-cells can be activated and differentiate to antibody-producing cells (103). In accordance with this, immunization of 2F5 KI mice with MPER peptide-liposome immunogens could rescue anergic B-cells to produce specific neutralizing antibodies (104, 105). More recently, a 2F5 germ-line KI mouse model showed 2F5 precursors deletion while the remaining anergic B cells could be also activated by germ-line mimicking immunogens (106). These outcomes indicated that the generation of 2F5 and 4E10 antibodies is likely controlled by immunological tolerance mechanisms and launched the hypothesis that HIV-1 host mimicry is an evolutionary strategy of pathogens and not particularly restricted to HIV-1 (95, 96). However, it is important to highlight that HIV-1 epitope mimicry does not impair the functionality of the host enzyme kynureninase, bound by 2F5 (107), and infusion of 2F5 or 4E10 in human recipients showed no major clinical complications (57), supporting the safety of eliciting MPER protective antibodies by vaccination (57, 107).

## The MPER Response during Natural Infection and Balance between Neutralizing and Non-Neutralizing Antibodies

The whole gp41 is mostly occluded by gp120 within the native viral spike, being the MPER transiently exposed during the fusion process (25). In consequence, B-cells accessibility to gp41 and native MPER may be compromised. Despite this, a strong antibody response is generated against the gp41 subunit in the course of HIV-1 infection probably due to gp120 shedding, non-functional forms of Env or transient epitope exposure during viral entry (108). Interestingly, the anti-gp41 humoral response can be detected two weeks after HIV-1 acquisition (108). This response, typically non-neutralizing and highly cross-reactive to gut commensal bacteria (92–94), is mainly focused against more exposed regions of gp41 such as the immunodominant disulfide loop, different from the MPER (108, 109).

Whereas MPER antibodies can be easily detected by ELISA, the analysis of their contribution to neutralizing activity of human plasma samples was found to be challenging. With this purpose chimeric SIV or HIV-2 viruses engrafted with HIV-1 MPER sequences or peptide-coated beads adsorption assays were developed (110–112). Accordingly, the presence of anti-MPER antibodies and the evaluation of their neutralizing capacity have been reported (35, 36, 65, 111, 113–116). The characterization of different cohorts in Europe, the United States, and South Africa indicated that MPER-specific neutralizing responses are less represented during natural infection comparing with other neutralizing specificities. For example, in a South African cohort of 156 HIV-1 infected individuals, only three showed higher titers of anti-MPER antibodies (65). Depletion of these antibodies resulted in loss of the neutralization breadth but the antibody specificities were found to be targeting a distinct epitope from those recognized by previously identified neutralizing epitopes (bound by 2F5 and 4E10 bNAbs), highlighting the existence of additional neutralizing specificities within the MPER (65). A recent study of the Protocol C cohort analyzed the neutralization profile of 439 plasma samples showing a far great less prevalence of MPER-specific antibodies when comparing with other specificities, mainly V3 N332-dependent glycan supersite (36). Remarkably, 27% of HIV-1 infected patients from an American cohort presented MPER-specific neutralizing activity (42). We previously showed that 66% of ART-naive chronically HIV-1 infected subjects presented MPER antibodies that were stable, at least for 1 year, and with an heterogeneous neutralizing capacity, highlighting the coexistence of neutralizing and non-neutralizing antibodies targeting the MPER (117). Moreover, anti-MPER antibodies correlate with the total anti-Env humoral response (117) and neutralization breadth (113, 118) and have been identified in HIV-infected individuals at different stages of the infection (119). Therefore, this landscape highlights that regardless of the cohort of study, anti-MPER antibodies (neutralizing and non-neutralizing) are present in HIV-1 infected subjects but their prevalence seems to be highly heterogeneous and probably strongly dependent on the methodology used (42, 65, 114, 117–119). Thus, the optimization of the current methodology for the quantification of MPER antibodies is highly desirable in order to establish their real prevalence. Human studies characterizing the MPER-specific neutralizing response are summarized in Table 1.

**Table 1:** Human studies detecting MPER-specific neutralizing responses.

Year published	Number of participants	Main findings	Reference
2006	96	One individual with 4E10-like neutralizing activity. No epitope competition	(110)
2007	3	No MPER-specific neutralizing activity	(112)
2007	14	4 individuals with MPER-specific neutralizing activity. 2 of them within the 6 months after seroconversion. No correlation with breadth	(111)
2009	156	3 individuals high MPER titer, associated with breadth. Distinct epitope from 4E10, 2F5, or z13	(65)
2009	70	MPER titer correlated with breadth. 4E10-like. Anti-cardiolipin antibodies correlated with breadth and MPER titer	(113)
2009	32	MPER-specific neutralization in 4 individuals	(114)
2010	19	Modest MPER-specific neutralization in 6 individuals	(35)
2011	308	4 out of 9 breadth neutralizers displayed MPER-specific neutralization (17–30% contribution)	(116)
2011	40	7 individuals > 40% breadth. MPER cross-neutralizing antibodies	(115)
2012	78	21 MPER-specific neutralizing activity. 8 out of 21 displayed 10E8 neutralization pattern	(42)
2014	35	8 individuals showed ID50 > 400 against chimeric HIV-2/MPER viruses whereas 66% had detectable MPER titers in ELISA and flow cytometry	(117)
2015	177	19% of the cohort showed MPER-specific neutralizing titers (ID50 > 1,000) against chimeric HIV-2/MPER viruses	(118)
2016	439	One individual with potent MPER-specific neutralizing activity	(36)

The results obtained from these studies also point out that the MPER is sufficiently immunogenic to generate a humoral response and that no specific constraints limit antibody generation against this region. However, the relatively low prevalence of MPER-neutralizing responses identified to date indicates that some hurdles are involved in the generation of this type of antibodies. The low accessibility of this region, which may compromise the affinity maturation process, as well as other mechanisms such as lipid cross-reactivity, might be determinant for the establishment of a balance between neutralizing and non-neutralizing MPER antibodies. Therefore, this balance is a relevant issue with important implications for vaccine design, where immunogens exposing native MPER neutralizing determinants should be implemented.

## Eliciting Anti-MPER Antibodies by Immunization

The particular features of the MPER described above, mainly low accessibility, close proximity to the membrane and subsequent hydrophobicity add additional hurdles for immunogen design against this vulnerability site. Moreover, the scarcity of MPER bNAbs isolated to date, comparing with other Env specificities does not contribute to enlarge our knowledge regarding the MPER complexity and the functional epitopes that should be targeted.

Initial approaches to induce 2F5 or 4E10-like antibodies attempted to introduce their corresponding binding sequences into chimeric viruses, fusion proteins or peptide-based vaccines (21). Although MPER-specific antibodies were elicited, neutralizing responses were not. Therefore, it became clear that additional variables beyond the recognition of specific peptidic sequences within the MPER should be considered. The common characteristics revealed later by MPER bNAbs, such as membrane cross-reactivity and binding to the gp41 prehairpin intermediate (72, 120), suggested that similar antibodies could be obtained by presenting MPER-based antigens in such precise conformational states in a membrane-like environment. In accordance, there are two major standpoints that are currently being addressed in MPER-based vaccinology: (i) what are the relevant structures that most likely mimic the native-bound form of MPER bNAbs and (ii) which is the role of membrane lipids over the MPER immunogenicity, including the precise lipid components and adjuvant systems. A summary of the most recent (since 2010) strategies followed in immunization protocols are listed in Table 2.

**Table 2:** Selection of recent immunization studies to elicit MPER neutralizing antibodies.

Immunogen	Animal model	Major findings	Reference
Prime/boost gp140 oligomer/MPER-peptide liposome	Guinea pig Rhesus macaque	Binding to the prefusion intermediate and the DKW 2F5 core	(121)
Liposomes containing a trimeric gp41-based protein	Llama	Bivalent single chain neutralizing antibody dependent of hydrophobic CDRH3	(87)
Fusion intermediate conformation of gp41 convalently linked to liposomes	Guinea pig	Gp41-specific antibodies binding to the gp41 fusion intermediate. Modest neutralization activity against 5 tier-1 and 2 tier-2 pseudovirus	(122)
Liposomes containing an MPER peptide, molecular adjuvants and encapsulated T-helper epitopes	Balb/c mouse	Superior antibody titers with MPER antigens anchored to liposomes comparing with oil-based emulsions	(123)
Proteoliposomes of diverse composition containing a gp41-based miniprotein	C57BL/6 mouse	Superior antibody titers of proteoliposomes based on lipids overrepresented on the viral membrane. Immunodominance against a 2F5 overlapping epitope	(124)
Recombinant Norovirus P particles (NoV PP) engrafted with the 4E10/10E8 epitopes emulsified with Freund’s adjuvant	Guinea Pigs Balb/c mouse	MPER-specific antibody titers and modest neutralization against SF162 isolate	(125)
MPER engrafted between the trimeric core structure and the trimeric domain of influenza A virus	Guinea pig	Induction of low MPER-specific titers	(126)
Bovine papilomavirus VLPs engrafted with the extended epitopes of 2F5 and 4E10, or the full MPER	Balb/c mouse	Epitope-specific IgG and mucosal secretory IgA	(127)
Engineered replication-competent reovirus vectors displaying the MPER sequence	Rabbit Balb/c mouse	No elicitation of MPER antibodies	(128)
Epitope-engrafted scaffold mimicking the 2F5-bound form of gp41	Guinea pigs Balb/c mouse	Isolation of antibodies resembling the 2F5 structure-specific recognition of gp41	(129)
Tandem peptide containing four copies of the 10E8 epitope with Freund’s Adjuvant	Rabbit	Modest neutralizing antiboy titers against tier-1 and tier-2 strains	(130)
Live attenuated *Salmonella* presenting the 10E8 epitope in the frimbriae	Balb/c mouse	MPER-specific antibodies and stimulated B-cell differentiation	(131)
Gp41 peptide grafted on virosomes	Rhesus macaque	Protection against SHIV challenge was correlated with the induction of vaginal gp41-specific IgA with transcytosis-blocking properties	(132)

Conformational states bound by anti-MPER bNAbs have been approached (121, 122, 129, 133). The use of computational methods permitted the design of scaffolds consisting in unrelated protein structures selected from database but able to accommodate the neutralizing 2F5 binding sequence in a conformation close to the peptide-bound crystal structure. Such scaffolds induced polyclonal responses mimicking a 2F5-like binding profile in immunized animals (129). Crystallographic analysis confirmed that monoclonal antibodies isolated from immunized animals mimicked the conformation of 2F5 in a flexible gp41 peptide, high affinity to the same sequence and similar angle of epitope approach (129, 134). Same outcomes were obtained with scaffolds targeting the 4E10 (135) and z13e1 (136) binding motifs. In spite of such structural mimicry, neutralizing activity was not achieved, likely because additional features such as membrane binding were not addressed in the design of these scaffolds.

Due to the importance for neutralization and their implication in a substantial portion of the free energy of 2F5, 4E10, and 10E8 binding, lipid-containing immunogen are important platforms being explored (71, 87, 88). Given that the complete epitope of anti-MPER bNAbs includes membrane components (74, 89) and that lipid recognition by CDR H3 impacts into their functionality (69, 72, 73, 86, 87), their potential for contributing to MPER-specific neutralizing responses by immunization is worth exploring. In this regard, membrane-mimicking platforms including viral-like particles (VLP) (137, 138) or liposomes (122–124) have been approached. It has been shown that membrane lipids can modulate the MPER structure likely by promoting a native-like conformation and demonstrated to improve immunogenicity (123, 124). In particular, we previously demonstrated that those lipids overrepresented in the viral membrane such as cholesterol and sphingomyelin have the potential to induce stronger antibody titers comparing with simple POPC lipids (124). Interestingly, MPER-specific antibodies from long-lived Bone marrow plasma cells from mice immunized with antigen-coupled liposomes have been also reported. Those antibodies showed that were shaped under selective pressure promoted by the MPER in the context of lipids and did not display any polyreactive feature (139).

Whereas the implementation of lipid-based platforms achieved MPER-specific antibodies, modest neutralizing titers have been reported by a few studies. For example, liposome-peptide antigens in combination with MPLA molecular adjuvant led to the isolation of two MPER-specific IgM antibodies showing lipid cross-reactivity but limited neutralizing capacity (140). The use of an HA/gp41 fusion protein in viral like particles induced modest 4E10-like neutralizating titers (141). One study by Dennison and colleagues obtained MPER-specific antibodies in NHP which bound preferentially to the gp41 prehairpin fusion intermediate rather than a recombinant gp41 construct by using a gp140 oligomer prime boosted with liposomes exposing an MPER peptide regimen. Such preferential binding was thought to be primarily due to structural modifications induced by the liposomes where the antigen was presented (121). Furthermore, the response mapped specifically the 2F5 DKW neutralizing core (121). In spite of these promising results, neutralizing activity was not achieved. Mimicking the gp41 prehairpin intermediate has been also approached by the design of a gp41 immunogen formulated in proteoliposomes. Immunization of guinea pigs showed modest neutralizing titers against tier 1 viruses, although the specificities responsible for such neutralization were not delineated (122). Finally, the role of non-neutralizing antibodies in protection has been shown in some studies. The presence of vaginal IgA with ADCC and transcytosis-bocking properties induced by gp41-grafted virosomes was associated with protection of NHP against SHIV challenge (132). Such vaccine platform was also evaluated in a Phase I clinical trial in healthy women. Vaginal secretions of vaccinated subjects were found to present transcytosis-blocking properties *in vitro* (142).

## Remark

In spite of the recent advances into the MPER physical and immunological properties, we still lack a full roadmap to generate a neutralizing response against this HIV-1 Env vulnerability site. The outcomes derived from MPER immunization studies clearly demonstrate that lipid cross-reactivity, binding to certain neutralizing epitopes or binding to gp41 native structures like the prehairpin intermediate are achievable. Although the implementation of these features will have a crucial role they will be likely insufficient to achieve the full properties of MPER-specific bNAbs in immunization protocols. In contrast, the selection of MPER non-neutralizing antibodies whose B-cell precursors may compete for the antigen presented cannot be excluded. While the knowledge gained from other Env vulnerability regions has advanced from the higher number of bNAbs isolated, to date only the potent 10E8 as well as 2F5 and 4E10 antibodies have been isolated. This fact highlights the need of the isolation of additional MPER bNAbs in order to bypass these gaps of our knowledge, improving immunogen design, while avoiding immunodominant non-neutralizing epitopes.

## Author Contributions

LM-A drafted the manuscript, JC reviewed the manuscript and JB and BC made substantial, direct, and intellectual contribution to he work. All authors approved it for publication.

## Conflict of Interest Statement

The authors declare that the research was conducted in the absence of any commercial or financial relationships that could be construed as a potential conflict of interest.
